# Interactions of immunological parameters among patients with periodontal disease

**DOI:** 10.1186/1939-4551-8-S1-A13

**Published:** 2015-04-08

**Authors:** Mohemid Al-Jebouri, Hadeel Al-Hadeethi

**Affiliations:** 1 College of Medicine,University of Tikrit, Iraq

## Background

Periodontal disease could be defined as a disorder of supporting structures of teeth, including the gingiva, periodontal ligament and alveolar bone. Complement and cytokines are believed to be important in periodontal infections .The understanding of interactions between different cytokines,immunoglobulins and complements may help to understand the response of gingiva to inflammation and also progression of gingivitis to periodontitis.

## Methods

118 samples were obtained from periodontal pockets after supragingival plaque was removed from the teeth to be collected. Determination of C3 ,C4 IgA, IgM, and IgG protein protein by radial immunodiffusion (RID) plate containing a specific antibody was used. IgE was determined by ST AIA PACK IgE II kit(Tosoh ,Japan) . Determination of human interleukins IL-4 and IL-10 in periodontal patient by quantikine ELISA kit was carried out(R&D system,USA).

## Results

It was found that a significant positive linear correlation between C3 and C4, C4 and IgA, and a significant negative correlation between C4 and IL10. The higher serum concentrations of IgA, IgM, IgG, IgE, C3, C4,IL-4,and IL10 was noticed among males than femals The comparison of serum IgE and C4 concentrations between males and females were statistically significant (p < 0.05) . While the comparison of serum IgG, IgM, IgA, C3 , IL-4,and IL-10 concentrations between males and females were statistically not significant ( p > 0.05) .

## Conclusions

The immunological analysis results showed that that the comparison of serum IgA, IgM and IgG ; C3 and C4 ; IL-4 and IL-10 concentrations between patients and control groups were significant ( p <0.05). It was found significant positive linear correlation between C3 and C4, C4 and IgA.

